# Hepatitis C Virus (HCV) Evades NKG2D-Dependent NK Cell Responses through NS5A-Mediated Imbalance of Inflammatory Cytokines

**DOI:** 10.1371/journal.ppat.1001184

**Published:** 2010-11-11

**Authors:** Damien Sène, Franck Levasseur, Michal Abel, Marion Lambert, Xavier Camous, Céline Hernandez, Véronique Pène, Arielle R. Rosenberg, Evelyne Jouvin-Marche, Patrice N. Marche, Patrice Cacoub, Sophie Caillat-Zucman

**Affiliations:** 1 Institut National de la Santé et de la Recherche Médicale (INSERM), U986, Hôpital St-Vincent de Paul, Paris, France; 2 Université Paris Descartes, Faculté de Médecine, Paris, France; 3 AP-HP, Hôpital Pitié-Salpêtrière, Département de Médecine Interne, Paris, France; 4 INSERM, U823; Université Joseph Fourier-Grenoble I, Faculté de Médecine, Institut Albert Bonniot, UMR-S823, Grenoble, France; 5 Université Paris Descartes, EA 4474 “Hepatitis C Virology”, Paris, France; 6 Université Pierre et Marie Curie, Faculté de Médecine, Paris, France; Nationwide Children's Hospital, United States of America

## Abstract

Understanding how hepatitis C virus (HCV) induces and circumvents the host's natural killer (NK) cell-mediated immunity is of critical importance in efforts to design effective therapeutics. We report here the decreased expression of the NKG2D activating receptor as a novel strategy adopted by HCV to evade NK-cell mediated responses. We show that chronic HCV infection is associated with expression of ligands for NKG2D, the MHC class I-related Chain (MIC) molecules, on hepatocytes. However, NKG2D expression is downmodulated on circulating NK cells, and consequently NK cell-mediated cytotoxic capacity and interferon-γ production are impaired. Using an endotoxin-free recombinant NS5A protein, we show that NS5A stimulation of monocytes through Toll-like Receptor 4 (TLR4) promotes p38- and PI3 kinase-dependent IL-10 production, while inhibiting IL-12 production. In turn, IL-10 triggers secretion of TGFβ which downmodulates NKG2D expression on NK cells, leading to their impaired effector functions. Moreover, culture supernatants of HCV JFH1 replicating Huh-7.5.1 cells reproduce the effect of recombinant NS5A on NKG2D downmodulation. Exogenous IL-15 can antagonize the TGFβ effect and restore normal NKG2D expression on NK cells. We conclude that NKG2D-dependent NK cell functions are modulated during chronic HCV infection, and demonstrate that this alteration can be prevented by exogenous IL-15, which could represent a meaningful adjuvant for therapeutic intervention.

## Author Summary

Natural killer (NK) cells are part of the innate immune response against virus infection. Their activation is the net result of signals emanating from a panel of inhibitory and activating receptors, among which the NKG2D activating receptor plays a major role. NKG2D ligands, the MHC class I related Chain (MIC) molecules, are induced on HCV-infected hepatocytes. In this paper, we show that NKG2D expression is decreased on NK cells from chronically infected HCV patients. As a consequence, NK cell cytolytic and IFNγ-producing functions are impaired. We show that this phenomenon is mediated by TGFβ produced by monocytes upon stimulation by the non-structural HCV-NS5A protein. NS5A could bind to TLR4 on monocytes, thus inducing the production of IL-10 and TGFβ, while inhibiting the production of IL-12. We further showed that TLR4-dependent IL-10 production by monocytes upon NS5A stimulation was mediated through the p38 and PI3 kinase pathways. In addition, we demonstrated that IL-15 could inhibit the TGFβ-mediated effects on NKG2D expression and NK cell functions. Collectively, these results identify a new dampening signal used by HCV to subvert innate immune response, and may provide new insights into the design of new strategies to restore NK cell functions in chronic hepatitis C.

## Introduction

Natural Killer (NK) cells are effectors of the rapidly acting antiviral innate immune system. They kill virally infected cells and are an important source of antiviral cytokines such as IFNγ. In addition, they establish an early and efficient dialogue with professional antigen presenting cells (APCs) that in turn, orchestrate the adaptive immune response towards Th1-type antiviral immunity [1]. NK cell activation is tightly regulated by the integration of signals emanating from a diverse array of inhibitory and activating receptors [2]. Inhibitory receptors, including Killer cell Immunoglobulin-like receptors (KIRs) and CD94/NKG2A, gauge expression of MHC class I molecules which can be compromised by viral immune subversion, and thus serves as an indicator of the integrity of cells. Activating receptors, including the natural cytotoxicity receptors (NCRs) and NKG2D, usually detect the presence of infectious non-self and/or stress-induced self ligands at the surface of infected cells.

Hepatitis C virus (HCV), which replicates in hepatocytes, mediates a chronic liver infection in the majority of infected individuals. NK cells abound in the normal liver, where they make up to 30% of resident hepatic lymphocytes [3]. This huge amount of NK cells in the liver suggests that they are important sentinel cells, surveying the liver for signs of damage or cellular stress. However, it also implies that HCV must divert NK cell-mediated responses in order to establish persistent infection. The importance of NK cells in the resolution of HCV infection is illustrated by the influence of genetic polymorphisms of KIR and their HLA ligands on the outcome of HCV infection [4]. Various alterations of NK cell phenotype have been described during chronic HCV infection, but results are often contradictory regarding the experimental conditions used (ex vivo or in vitro cytokine-stimulated), the modifications involved and their consequences on effector functions [5], [6], [7], [8], [9], [10], [11].

The NKG2D activating receptor is constitutively expressed on human NK and CD8 T cells [12]. Its ligands, the MHC class I chain-related A and B proteins (MICA and MICB) and UL-16 binding proteins (ULBP1–4), are almost undetectable in normal tissues, but are induced on the cell surface by various stresses such as DNA damage, tumor transformation and intracellular infection. The importance of the NKG2D defense system is highlighted by the observation that tumors and viruses have developed several mechanisms for evading NKG2D-mediated recognition [13], [14], [15], [16], [17]. The overall contribution of the NKG2D pathway in the control of HCV infection is unclear [7], [10]. We show here that NKG2D is downmodulated on circulating NK cells, and consequently NK cells are functionally impaired. This defect is mediated by the HCV-NS5A protein, which disturbs the equilibrium between pro- and anti-inflammatory monocyte-derived cytokines.

## Results

### NKG2D expression is decreased on circulating NK cells during chronic HCV infection

**Figure 1:**
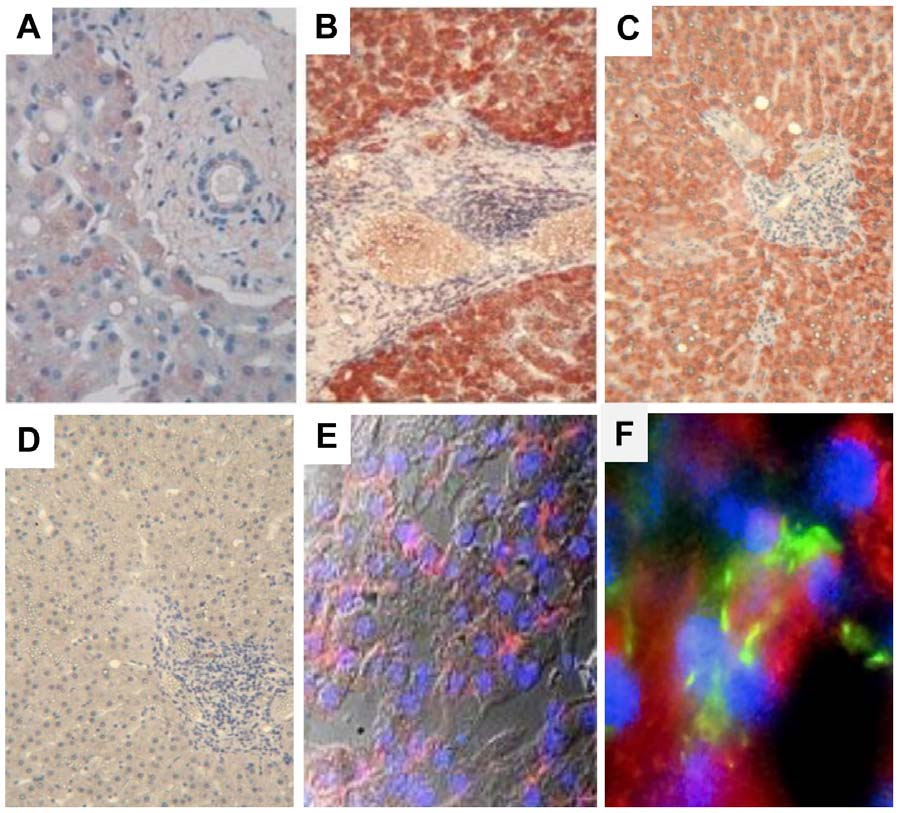
MIC proteins are overexpressed in HCV-infected liver. Staining for MIC proteins in sections of paraffin-embedded (A–D) or cryopreserved (E, F) liver biopsies from HCV-negative control (A) or chronically HCV-infected patients (B–F). Control isotype is shown in (D). A strong and diffuse expression of MIC (red) is observed in hepatocytes from HCV-infected patients, in the cytoplasm (B, C) and at the membrane (E, confocal microscope analysis). MIC expression is seen in HCV infected cells (NS5A staining in green) and in some adjacent cells (F). Nuclei appear colored in blue. Results are representative of 12 patients for paraffin-embedded biopsies, and 3 patients for cryopreserved biopsies. Original magnification, ×10 (A, D), ×40 (E) and ×60 (F).

MIC proteins are induced at the cell surface upon exposure to various pathogens [11], [18], [19], [20], serving as a warning signal that alerts NK cells to mediate effector functions through NKG2D signaling. We thus examined if MIC was expressed in the liver during chronic HCV infection. While staining of control livers showed a faint expression of MIC in the cytoplasm of some hepatocytes and Kupffer cells only, HCV-infected livers displayed a strong and diffuse expression of MIC in the cytoplasm and at the surface of HCV-infected hepatocytes, and also in some uninfected hepatocytes and large mononuclear cells in portal spaces resembling macrophages (Figure 1).

**Figure 2:**
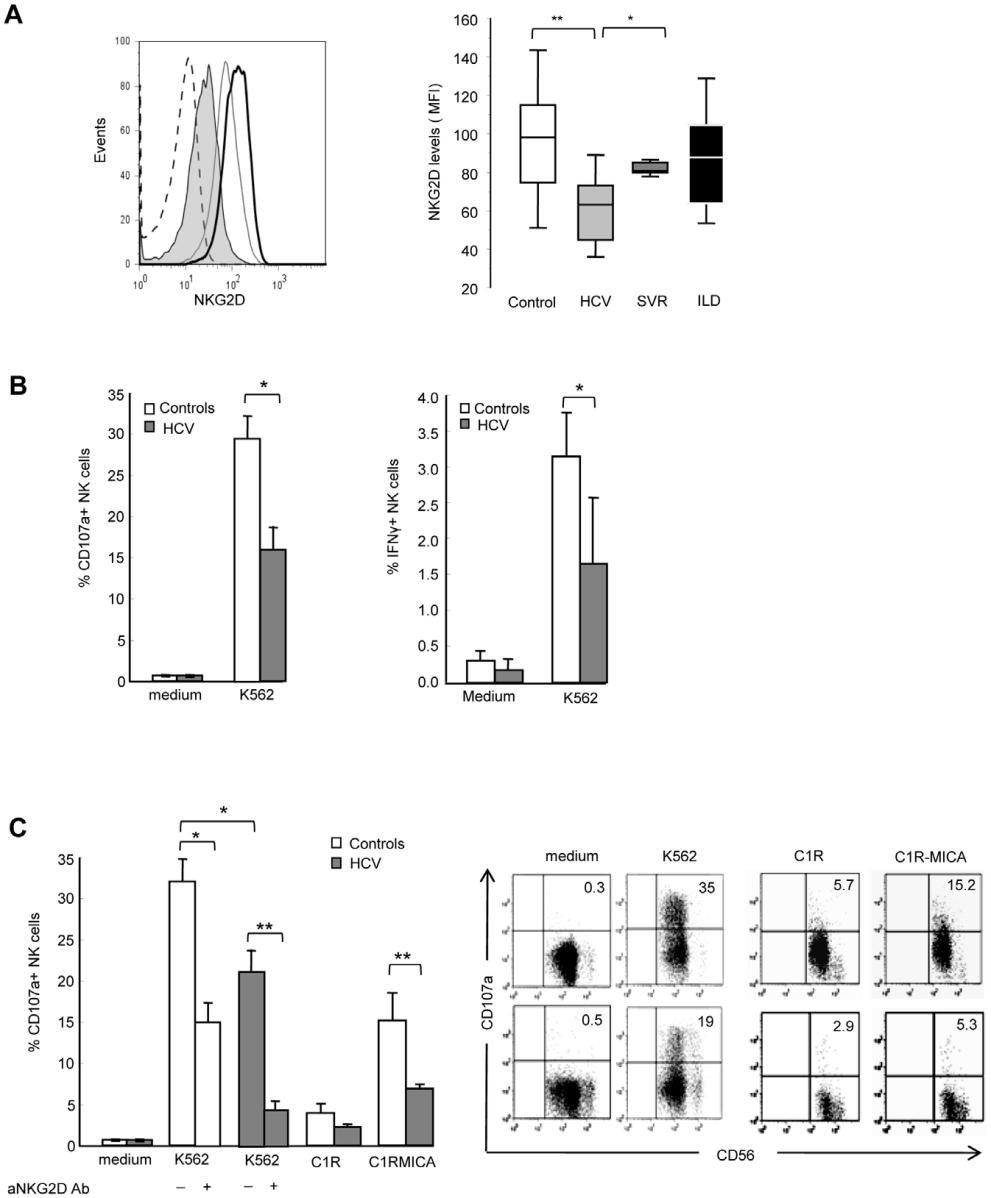
NKG2D levels and NK cell functions are altered in HCV-infected patients. A) Freshly isolated PBMCs were stained with CD56, CD3 and NKG2D mAbs, and analyzed by FACS. Gating on CD3-CD56+ cells identified NK cells. *Left panel*: Representative NKG2D staining in one healthy control (dark line), one chronically-infected HCV patient (plain gray) and one HCV patient with sustained viral response (SVR, empty gray). Isotype control is shown in dotted line. *Right panel*: Combined data showing the mean NKG2D levels on NK cells from 23 controls, 41 HCV patients, 9 patients with SVR, and 9 patients with non-infectious inflammatory liver disease (ILD). Data are expressed as box plots, in which the horizontal lines illustrate the 25^th^, 50^th^, and 75^th^ percentiles of the MFI of NKG2D. The vertical lines represent the 10^th^ and 90^th^ percentiles. * *P*<10^−4^, ** *P*<10^−3^. B) Frequency of CD107a+ degranulating (*left panel*) or IFNγ-producing (*right panel*) NK cells incubated for 6 hr in medium alone, or in the presence of MHC class I-negative K562 cells (E∶T ratio 1∶1). Results show the mean ± SEM values in 11 HCV patients and 15 healthy controls. * P = 0.003. C) Frequency of CD107a+ NK cells incubated for 6 hr with K562 cells (alone or in the presence of 20 µg/ml of NKG2D blocking mAb or control IgG), C1R cells or C1R-MICA transfectants. E∶T ratio 1∶1. *Left panel*: Combined data in 6 HCV patients (grey bars) and 6 healthy controls (white bars). * P = 0.003, ** P = 0.0001. *Right panel*: Dot plots show a representative experiment in one control (upper line) and one HCV patient (lower line). Numbers in the quadrant indicate the percentage of CD107a+ NK cells.

NKG2D is constitutively expressed on NK cells, and should therefore mediate recognition and destruction of MIC-expressing cells. Due to the restricted availability of fresh HCV-infected liver samples to isolate infiltrating NK cells, we examined the expression of NKG2D on circulating NK cells. No significant difference in the percentage of circulating NK cells, or in the proportion of CD56^bright^/CD56^dim^ cells was detectable between patients and controls (data not shown). The percentage of NKG2D-expressing NK cells was similar in HCV patients and healthy controls (>95% of NK cells in both groups). However, a decreased expression of NKG2D on both CD56^bright^ and CD56^dim^ NK cells was detected in HCV viremic patients as compared with healthy controls (mean MFI: 61±15 versus 93±25, P<10^−4^), HCV patients with sustained viral response (SVR) after treatment (81±14, P<10^−3^) or patients with non-infectious chronic inflammatory liver disease (87.4±24.5, P<10^−3^) (Figure 2A). Although showing some variability among viremic patients, NKG2D levels were not correlated with age, sex, HCV viral load, genotype, ALT levels, liver fibrosis or activity score.

To evaluate the functional consequences of this NKG2D reduction, we quantified NK cell IFNγ production and CD107a degranulation by flow cytometry. Freshly purified circulating NK cells from HCV patients showed impaired IFNγ production in the presence of MHC class I-negative K562 as compared to NK cells from healthy controls. In addition, NK cells from HCV patients showed a two-fold decreased degranulation in the presence of K562 targets (mean CD107 expression 29.3%±2.7 in controls compared to 15.9%±2.9 in patients, P = 0.003) (Figure 2B). This defective NK cell function was at least in part dependent on NKG2D, as shown using C1R-MICA as target cells. CD107a expression on NK cells positively correlated with NKG2D levels (Spearman rho (*r*)  = 0.62, P = 0.008, Supplementary Figure S1). Moreover, NKG2D blocking by anti-NKG2D antibody largely inhibited inhibited NK cell degranulation in both HCV patients and healthy controls (Figure 2C). That NK cell degranulation was not fully abrogated indicates however, that it likely involves other activating receptor(s) in addition to NKG2D. Altogether, these results suggest that the signaling pathway initiated by NKG2D on target exposure may not operate properly in HCV patients due to NKG2D reduction on NK cells.

### Soluble TGFβ is associated with NKG2D reduction

**Figure 3:**
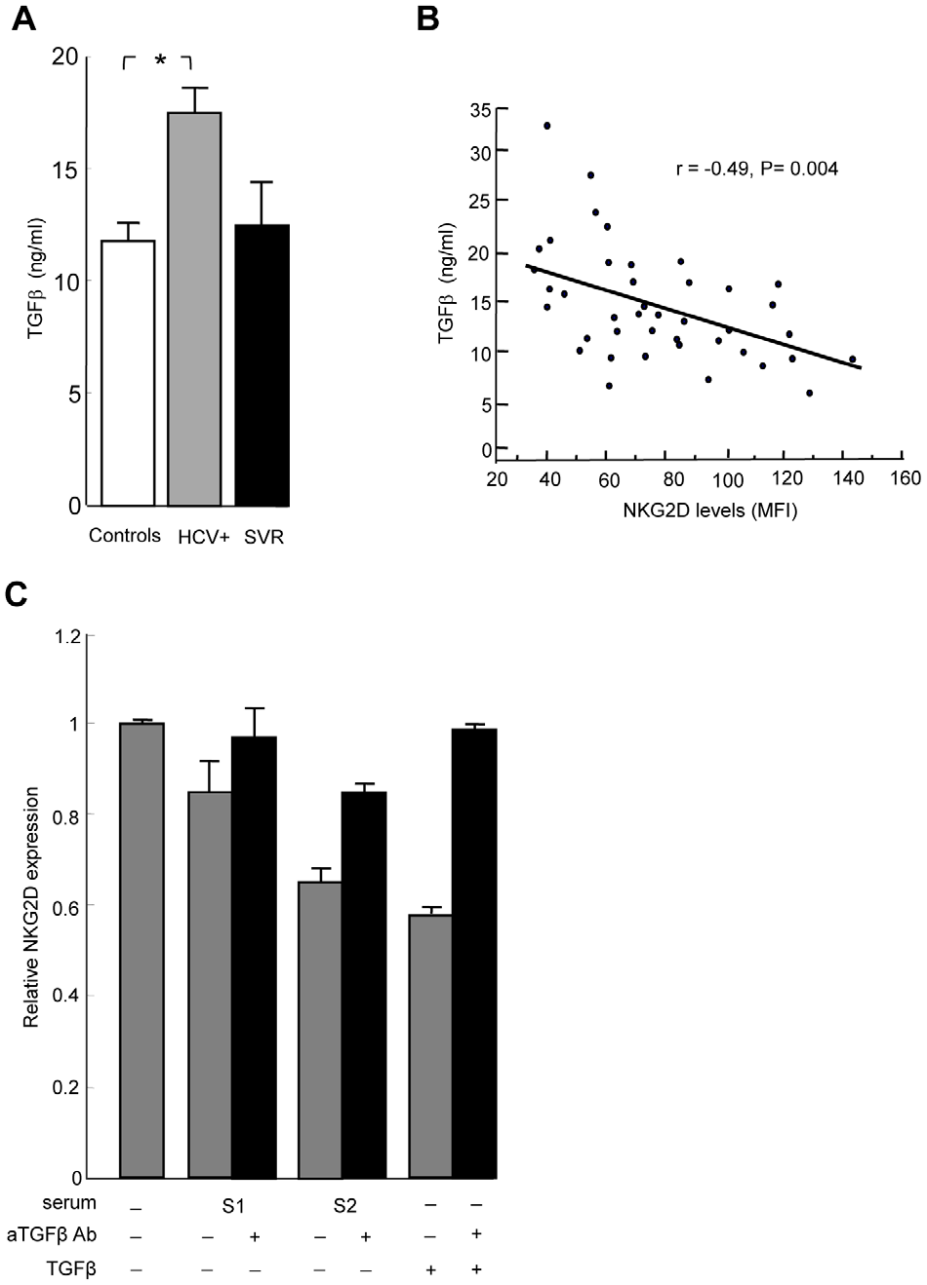
TGFβ is overproduced in HCV patients, and mediates NKG2D reduction on NK cells. A) The levels of total TGFβ were significantly higher in serum from 34 HCV patients compared to 23 controls (* P = 0.008). Only 4 samples from SVR patients could be analyzed. B) Inverse correlation between serum TGFβ levels and NKG2D levels on NK cells in HCV patients. Pearson's correlation coefficient r = −0.49, P = 0.004. C) NKG2D levels are reduced in a TGFβ-dependent manner. NK cells were cultured in medium alone, or in medium containing 10% serum from representative HCV patients with known TGFβ concentration (S1: 6 ng/ml, i.e. 0.6 ng/ml final concentration; S2: 32 ng/ml, i.e. 3.2 ng/ml final concentration), or in medium containing 10 ng/ml of recombinant TGFβ. NKG2D expression was analyzed 24 hr later by flow cytometry. Addition of 10 µg/ml of neutralizing anti-TGFβ mAb during incubation largely reversed NKG2D reduction. Results are expressed as NKG2D levels on NK cells cultured in each indicated condition relative to the levels on NK cells cultured in medium alone, and represent mean ± SEM of 2 independent experiments. P = 0.02 for all comparisons (Kruskall Wallis test).

Systemic NKG2D downregulation on immune effector cells has been related to the release of soluble factors such as MIC molecules (sMIC) in the serum of cancer patients [13]. We measured sMIC in the serum of HCV patients and healthy controls, and found similar low levels of sMIC in both groups (data not shown). TGFβ is another mediator of systemic NKG2D downregulation [21], [22], [23]. Total TGFβ levels were higher in the serum of HCV-infected patients compared to controls and SVR patients (Figure 3A). An inverse linear relationship was observed between TGFβ and NKG2D levels on NK cells (Figure 3B). To investigate whether serum of HCV patients could mimic the effect of exogenous TGFβ on NKG2D expression, we incubated control NK cells with recombinant TGFβ or with serum from representative HCV patients with known TGFβ concentration, and analyzed NKG2D expression. NKG2D levels were reduced in a TGFβ concentration-dependent manner, and were largely restored when incubation was performed in the presence of neutralizing anti-TGFβ antibody (Figure 3C).

### TGFβ is secreted by monocytes upon stimulation by the HCV-NS5A protein

**Figure 4:**
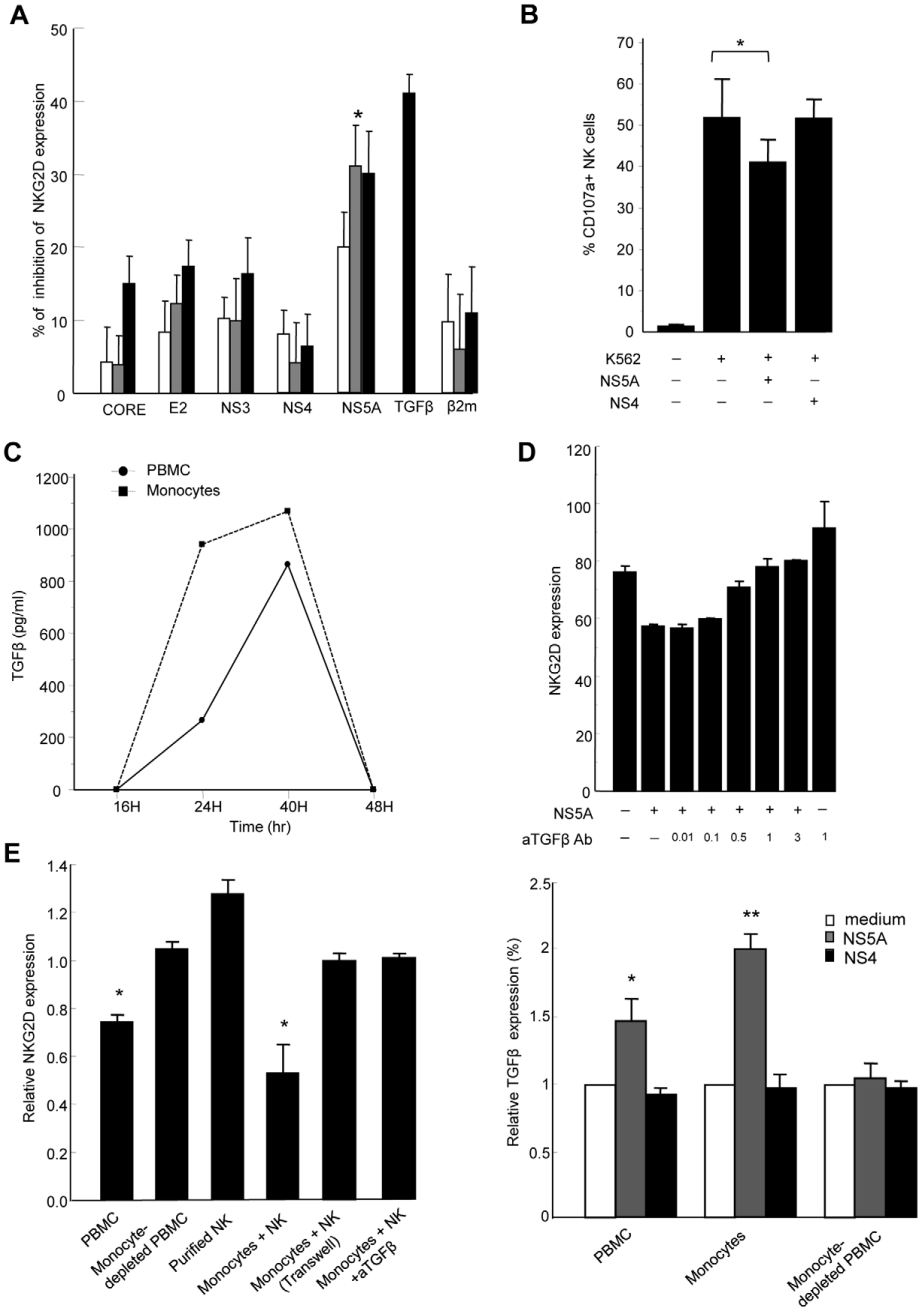
HCV-NS5A protein mediates TGFβ-induced downmodulation of NKG2D. A) Control PBMCs (0.2×10^6^/ml) were stimulated with HCV-core, -E2, -NS3, -NS4, -NS5A or β2 microglobulin proteins at 0.1 µg/ml (white bars), 0.5 µg/ml (gray bars), or 1 µg/ml (black bars), or with recombinant TGFβ (10 ng/ml) for 48 h, and NKG2D expression was analyzed on CD3-CD56+ NK cells. Results are expressed as the percentage of inhibition of NKG2D expression in each condition relative to medium alone. Mean ± SEM of 3 independent experiments are shown. * P = 0.001. B) NS5A-mediated decrease in NK cell lytic potential. Control PBMCs were exposed to NS5A, NS4 or medium alone for 48 h, after which NK cell lytic potential to K562 target cells was evaluated by flow cytometry (CD107a expression). * P = 0.008. C) Kinetic analysis of TGFβ secretion upon 48 h stimulation of PBMCs (circles) or purified monocytes (squares) with 0.5 µg/ml of NS5A. TGFβ concentrations are given after background subtraction (cells cultured in medium alone). D) Pretreatment of PBMCs with increasing (0.01–3 µg/ml) concentrations of neutralizing TGFβ mAb prior to incubation with NS5A induces a dose-dependent increase of NKG2D on NK cells. E) Monocytes are the source of TGFβ upon NS5A stimulation. *Left panel*: The indicated cell populations were stimulated with 0.5 µg/ml of NS5A for 40 hr after which NKG2D expression was evaluated on NK cells. Results are expressed as NKG2D levels on NK cells in the indicated condition relative to the values in medium alone. NK cells were cultured with purified monocytes at the ratio of 1∶1 with or without Transwell (0.4 µm) separation or in the presence of 10 µg/ml anti-TGFβ mAb or isotype Ab. Data are mean ± SEM of 3 independent experiments. * P<0.002 for comparison with the values in medium alone. *Right panel*: Supernatants were harvested and assayed for TGFβ content. Values are normalized to TGFβ produced by cells cultured in medium alone and represent the mean ± SEM of 4 independent experiments. * P = 0.006, ** P = 0.0003.

Engagement of the HCV receptor CD81 by the major HCV envelope protein E2 was shown to block NK cell functions triggered by NKG2D engagement [24]. We thus hypothesized that HCV-E2 might modulate NKG2D expression. PBMCs from normal donors were exposed to recombinant HCV-E2, as well as to other structural and non-structural HCV proteins for 6 to 48 hr, and NKG2D levels were measured on NK cells (Figure 4A). While HCV-E2, -core, -NS3, or -NS4 proteins had no or minor effect, HCV-NS5 induced a dose-dependent reduction of NKG2D on NK cells, which was maximal at 48 hr. Using different recombinant NS5A preparations (E. Coli-derived full length NS5, yeast-derived NS5 2054-2995 or E. Coli-derived NS5A amino acid 2061–2392), we reproducibly identified the NS5A protein as being responsible for this effect. At a concentration of 0.5 µg/ml, NS5A reduced NKG2D expression by 40% (P = 0.001), which was of the same order of magnitude as 10 ng/ml of recombinant TGFβ used as positive control. Of note, NS5A stimulation also induced downmodulation of the NKp30 activating receptor (Supplementary Figure S2), in line with the previously described effect of TGFβ on NKp30 expression [23]. The β2-microglobulin, used as control for irrelevant protein produced in E. Coli, had no effect on NKG2D expression. All recombinant proteins used were tested for the absence of significant lipopolysaccharide (LPS) contamination (0.054 endotoxin unit/µg recombinant protein in the case of NS5A, i.e. 5.4 pg/µg protein). In addition, pretreating NS5A by 10 µg/ml of polymyxin B was without effect, ruling out the possibility of contaminating LPS being the factor responsible for NKG2D reduction. Inactivation of the NS5A protein by freeze/thaw before incubation with PBMCs abolished the NS5A-mediated NKG2D reduction, suggesting that it required intact protein conformation.

To verify that NS5A-mediated downregulation of NKG2D on NK cells was accompanied by a decrease in their functional capacity, PBMCs were exposed to NS5A, NS4 or medium alone for 48 h, after which NK cell degranulation capacity in the presence of K562 target cells was evaluated by flow cytometry. The presence of NS5A in PBMC culture induced a significant decrease of CD107a expression on NK cells, while NS4 had no effect, confirming that NS5A is responsible for a decreased functionality of NK cells (Figure 4B).

We then measured the TGFβ concentration in culture supernatants from PBMCs exposed to NS5A or medium alone for 12 to 48 h. Levels of TGFβ progressively increased in the presence of NS5A (Figure 4C). To confirm that the NS5A effect was indeed related to TGFβ production, we pretreated PBMCs with anti-TGFβ antibody prior to stimulation with NS5A. Blocking TGFβ abrogated the NS5A-induced reduction of NKG2D on NK cells in a dose-dependent way (Figure 4D).

When similar experiments were performed on freshly purified NK cells, NS5A stimulation failed to downmodulate NKG2D, suggesting that TGFβ was likely produced by a distinct cell population among PBMCs. To identify the source of TGFβ, we cocultured purified NK cells with different components of autologous PBMCs, including adherent or non-adherent cells, monocyte-depleted PBMCs, or purified monocytes. Cells were stimulated for 6–48 hr with NS5A, after which NKG2D was measured on NK cells. At the same times, culture supernatants were recovered and were assayed for TGFβ production. As shown in Figure 4E, only monocyte-containing populations induced a significant decrease of NKG2D expression on NK cells (p<0.002). Notably, downregulation of NKG2D was completely lost in the Transwell system, indicating that monocyte-NK cell contacts were required for this effect. NKG2D reduction was accompanied by an increased production of TGFβ in cell supernatants, which was maximal after 40 hr of NS5A stimulation (Figure 4C). In addition, neutralization of TGFβ in the coculture system of monocytes and NK cells fully restored NKG2D expression on NK cells. Altogether, these results indicate that NS5A protein induces TGFβ production by monocytes, which in turn affects NKG2D expression and inhibits NK cell functions.

### NS5A augments IL-10 and suppresses IL-12 production by monocytes

**Figure 5:**
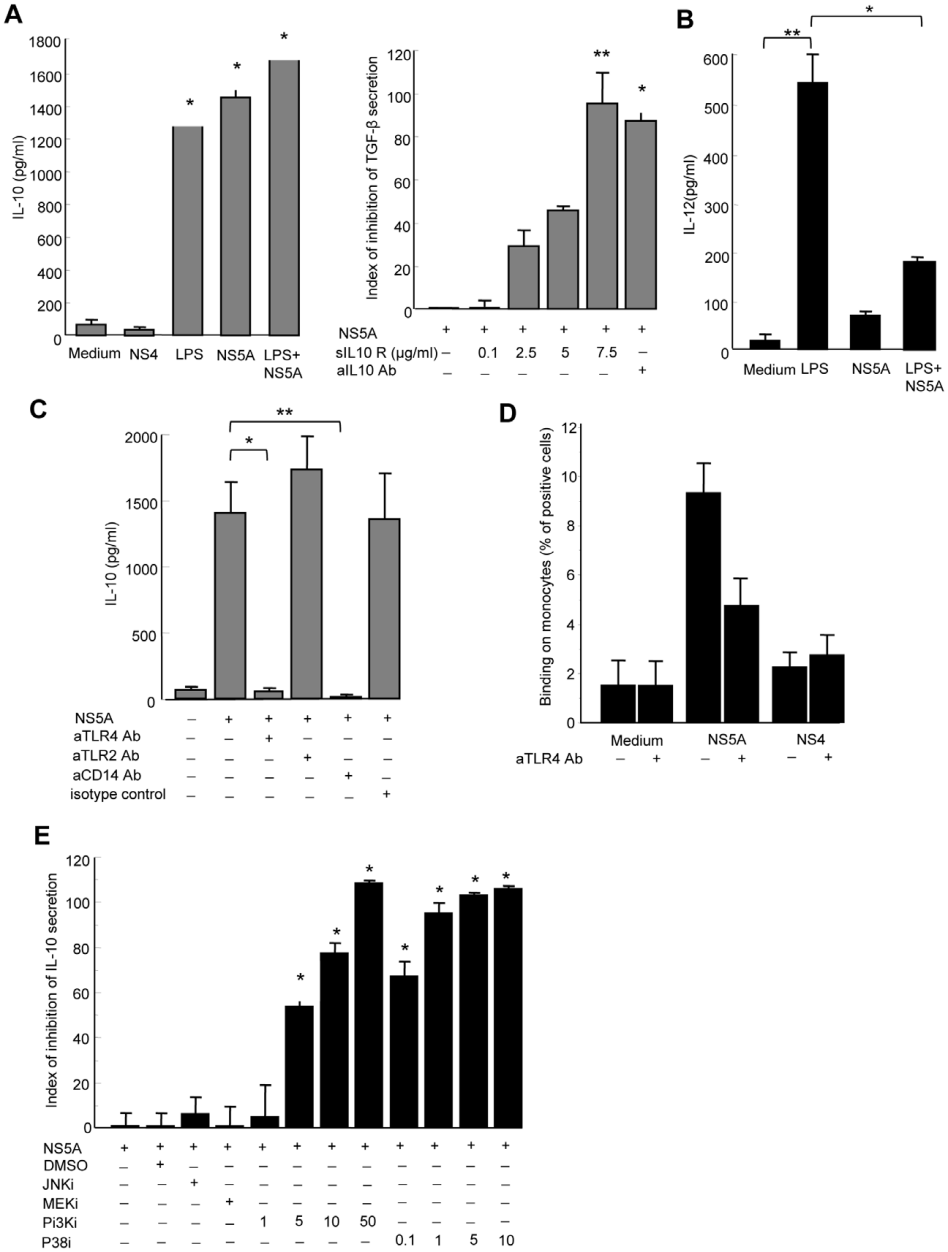
NS5A induces IL-10 and suppresses IL-12 production by monocytes through TLR4-mediated activation of p38 and PI3 kinase signaling. A) *Left panel*: Control monocytes (0.1×10^6^/ml) were cultured in medium alone, or in the presence of NS4 (0.5 µg/ml), NS5A (0.5 µg/ml), LPS (1 µg/ml), or NS5A+LPS for 24 hr, and cell supernatants were analyzed for IL-10 by ELISA. Data are expressed as the mean ± SEM of three independent experiments. * P = 0.01. *Right panel*: Monocytes were pretreated with IL-10 neutralizing mAb or soluble IL-10 receptor prior to stimulation with NS5A, and TGFβ levels in the culture supernatants were quantified by ELISA. Results are expressed as index of inhibition of NS5A-mediated TGFβ levels, calculated as: [(TGFβ (NS5A) - TGFβ (NS5A+mAb))/TGFβ (NS5A) - TGFβ (medium)]x100, and are the mean ± SEM of three independent experiments. * P = 0.02, ** P = 0.0005. B) For IL-12p70 production, monocytes were first primed by IFNγ for 16 h and then stimulated with LPS (1 µg/ml), HCV-NS5A (0.5 µg/ml) or both for 24 hr, after which supernatants were analyzed for IL-12 by ELISA. Data are expressed as the mean ± SEM of three independent experiments. * P = 0.02, ** P = 0.001. C) Purified monocytes were pretreated with 5 µg/ml of anti-TLR4, -TLR2 or -CD14 neutralizing antibodies prior to 24 hr stimulation with 0.5 µg/ml of NS5A, after which IL-10 levels in the supernatants were measured by ELISA. Results are the mean ± SEM of IL-10 levels from mean replica values in 3 independent experiments. * P = 0.02, ** P = 0.01. D) Binding of NS5A to monocytes. Control monocytes were incubated for 30 min at 4°C with 0.5 µg/ml of recombinant NS5A, NS4 or medium alone. Binding of NS5A to monocytes was revealed by staining with anti-NS5A 9E10 antibody and flow cytometry analysis. Results are expressed as the percentage of positive cells, and are the mean of 4 independent experiments. E) Monocytes were left untreated, or treated with JNK inhibitor (SP600125, 10 µM), MEK inhibitor (U0126, 10 µM), PI3K inhibitor (LY294002, 1–50 µM), p38 inhibitor (SB203580, 0.1–10 µM) or DMSO alone as negative control, for 1 hr prior to stimulation with NS5A (0.5 µg/ml) for additional 24 h. Supernatants were assayed for IL-10 content by ELISA. Results represent the percentage of inhibition of IL-10 production, and are the mean ± SEM of 3 separate experiments. * P<0.01.

Activation of monocytes by microbial products usually induces the production of IL-12, and to a lesser extent of IL-10. We thus wondered if the ability of NS5A to regulate the production of TGFβ was more global, and measured the production of IL-10 and IL-12 in supernatants from control monocytes stimulated for 24 to 48 h with NS5A, NS4, medium alone, or LPS as a positive control. High levels of IL-10 (>1400 pg/ml) were detected 24 hr after NS5A stimulation (Figure 5A, left panel). These levels were of the same order of magnitude as those induced by 1 µg/ml of LPS. We then wondered if NS5A-induced TGFβ production was related to autocrine IL-10 release. Blocking IL-10 or its receptor abrogated the NS5A-induced TGFβ secretion in a dose-dependent manner, but did not modify the basal TGFβ production by monocytes (Figure 5A, right panel). Furthermore, we found elevated IL-10 levels in the sera of HCV patients, that were positively correlated with TGFβ levels (r = 0.39, P = 0.016).

By contrast, very low amounts of IL-12 were detected in supernatants of NS5A-stimulated monocytes, while LPS induced high levels of IL-12 as expected (Figure 5B). We thus hypothesized that NS5A might inhibit the LPS-induced production of IL-12 by monocytes. Indeed, pretreatment of monocytes by NS5A strongly inhibited IL-12 production upon LPS stimulation.

Taken together, these findings demonstrate that NS5A potently increases the production of anti-inflammatory cytokines IL-10 and TGFβ, while concurrently suppressing the production of proinflammatory IL-12. The lack of NS5A-induced IL-12 secretion also confirms that LPS contamination is not responsible for the NS5A-mediated effect in monocytes.

### NS5A interaction with TLR4 instructs monocytes to preferentially induce IL-10 secretion

NK cell activation requires signals provided by APCs that sense pathogen products through conserved pattern-recognition receptors such as Toll-like receptors (TLRs). In particular, TLR2 and TLR4 are involved in extracellular sensing of several viral proteins by monocytes and dendritic cells [25], [26], [27]. We thus hypothesized that NS5A might interact with TLR2 or TLR4 signaling in monocytes. Pretreating monocytes with blocking anti-TLR4 antibody, or with antibody to the TLR4 associated molecule CD14, fully abolished NS5A-mediated IL-10 production, while blocking anti-TLR2 antibody had no effect (Figure 5C). This suggested that NS5A might interact with TLR4 on monocytes. To support this hypothesis, freshly purified monocytes were incubated at 4°C with NS5A or NS4, and binding was revealed by staining with anti-NS5A antibody and flow cytometry analysis. A significant binding of NS5A on monocytes was observed, that was inhibited by almost 50% in the presence of blocking anti-TLR4 antibody (Figure 5D).

TLR4 signaling results in the downstream activation of NF-kB, MAPK (p38 and JNK) and PI3K pathways[28]. TLR4 activation may contribute to IL-10 production via p38 and PI3K [29], while PI3K is an endogenous suppressor of IL-12 production triggered by TLR4 [30]. We pretreated monocytes with pharmacological inhibitors of signaling molecules prior to stimulation with NS5A, and measured IL-10 production. Inhibition of p38 or PI3K suppressed NS5A-induced production of IL-10 in a dose-dependent way, while other inhibitors had no or minor effects (Figure 5E).

Altogether, our results indicate that, upon NS5A interaction with TLR4, monocytes preferentially secrete IL-10 through activation of the p38 and PI3 kinase pathways, but are prevented from secreting IL-12.

### NS5A mediates the NKG2D-downregulating effect in supernatants of HCV-infected cells

**Figure 6:**
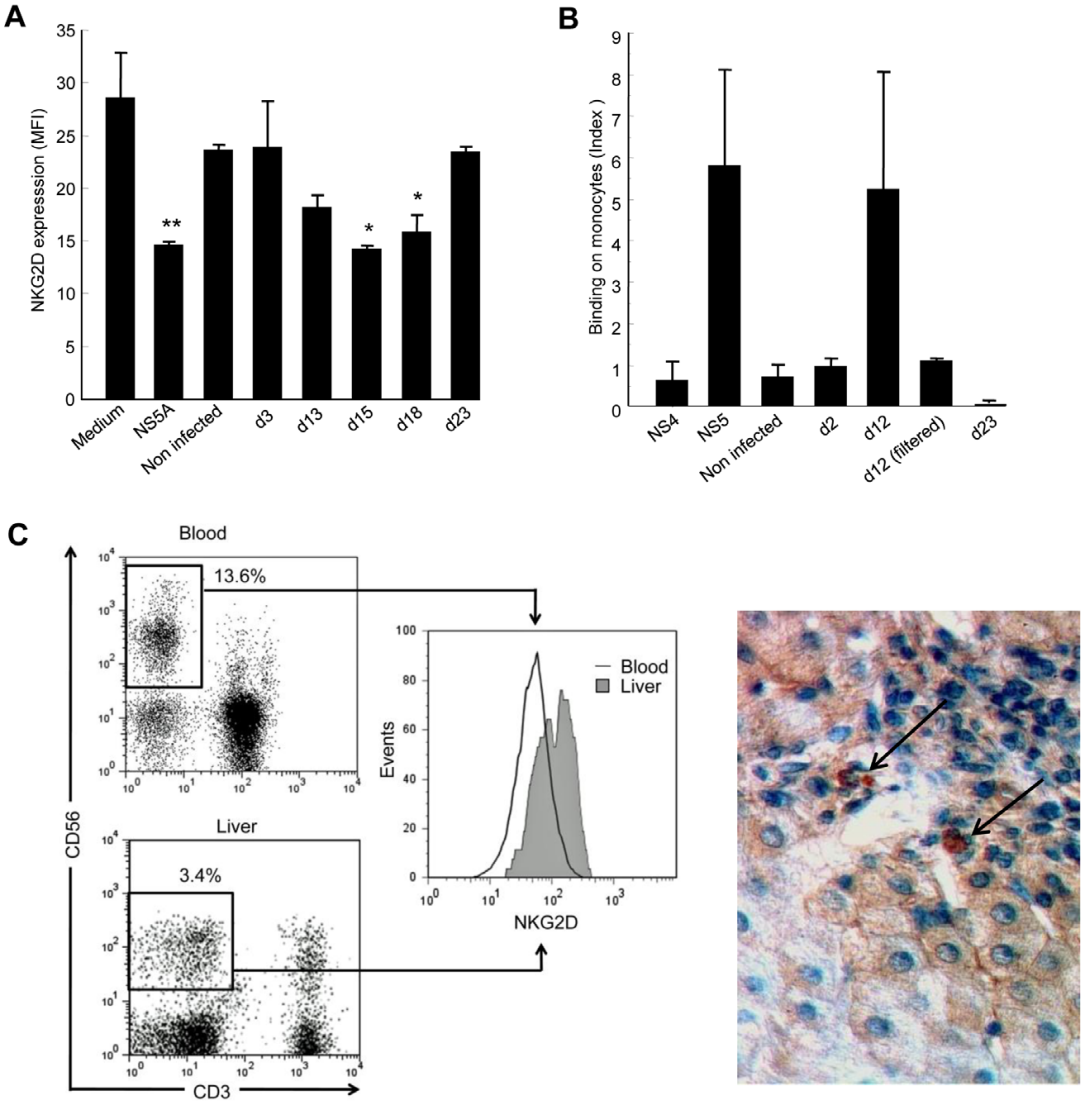
Supernatants of HCV-infected Huh-7.5.1 cells reproduce the effect of recombinant NS5A. A) Control PBMCs were cultured for 48 hr in medium alone, in medium containing 0.5 µg/ml of recombinant NS5A, or in medium containing 50% of supernatant from non-infected or JFH1-replicating Huh-7.5.1 cells (collected at day 3, 13, 15 or 18 post transfection). NKG2D expression on NK cells was analyzed 48 hr later by flow cytometry. * P<0.05, ** P = 10^−4^ for comparison with the values in medium alone. B) Control monocytes were incubated for 30 min at 4°C with supernatants from non-infected or JFH1-infected Huh-7.5.1 cells collected at the indicated day post infection, or with 0.5 µg/ml of recombinant NS4 or NS5A proteins as negative and positive controls, respectively. Binding of NS5A to monocytes was revealed by staining with anti-NS5A 9E10 antibody and flow cytometry analysis. Results are expressed as the index of binding ([% binding with culture supernatant - % binding with medium alone]/% binding with medium alone), and represent mean ± SEM of 2 independent experiments. C) NKG2D expression on liver-infiltrating NK cells from HCV patients. *Left panel*: Representative NKG2D staining in paired circulating and liver-infiltrating NK cells freshly isolated from one chronically infected HCV patient. Dot plots and corresponding histograms show the frequency of NK cells (boxed CD3-CD56+) and their NKG2D expression. *Right panel*: NKG2D staining with anti-NKG2D mAb in liver biopsy from a chronically HCV-infected patient. Results are representative of 4 HCV patients. Only few NKG2D-positive cells (arrows) are found within a mononuclear infiltrate surrounding a portal tract. Original magnification, ×40.

NS5A is not found in viral particles secreted by infected cells, which raises the question of its availability in the extracellular medium. However, it is becoming increasingly clear that HCV infection of hepatocytes has direct cytopathic effects, suggesting that NS5A might be released from apoptotic/necrotic infected cells [31], [32], [33]. To verify this hypothesis, we determined whether supernatants of HCV-infected cells induced downmodulation of NKG2D. To this aim, we used Huh-7.5.1 hepatoma cells transfected or not with the infectious genotype 2a JHF1 replicon [34], [35]. Control PBMCs were cultured for 48 h with Huh-7.5.1 culture supernatants or with recombinant NS5A as positive control, after which NKG2D levels on NK cells were measured (Figure 6A). Supernatants from Huh-7.5.1 uninfected cells, or those recovered as soon as day 3 post transfection, did not modify NKG2D levels. By contrast, supernatants recovered on days 13, 15 and 18 post transfection reproducibly induced downmodulation of NKG2D at levels similar to those obtained using 0.5 µg/ml rNS5A. At these time points, the majority of cells in the culture expressed HCV proteins, infectivity titers in culture supernatant were maximal, and cytopathic effects were observed, as reported [31]. Notably, there was no effect of supernatants recovered at day 23, i.e. at a time cells were cytologically normal and levels of extracellular infectious virus had declined.

To rule out the possibility that this effect was mediated by TGFβ, we quantified TGFβ in Huh-7.5.1 culture supernatants. In all conditions studied, TGFβ levels were below 40 pg/ml and could thus not be responsible for the observed NKG2D-downregulating activity. Taken together, these data indirectly suggested that NS5A is released by HCV-replicating cells, most likely among cell debris generated by infection.

Since we previously observed a specific binding of recombinant NS5A on monocytes, we incubated control PBMCs with supernatants from uninfected or infected Huh-7.5.1 cells, or with recombinant NS5A or NS4 proteins, after which NS5A binding to monocytes was evaluated by staining with anti-NS5A antibody. Supernatants from uninfected cells, or supernatants recovered on days 2 and 23 post transfection did not show any binding signal, in accordance with their lack of NKG2D-downregulating activity. However, supernatants recovered on day 12 post transfection gave a binding signal that was of the same order of magnitude than that observed with recombinant NS5A (Figure 6B). Notably, this effect was abrogated when day 12 supernatants were filtered in order to eliminate cell debris, suggesting that NS5A was not released from JHF1-replicating Huh-7.5.1 cells in a soluble form, but was rather associated with apoptotic-cell components. Our attempts to corroborate the presence of NS5A in day 12 supernatants using ELISA or Western blot techniques, or to deplete NS5A from these supernatants using anti-NS5A antibody, were unsuccessful (data not shown). This could be due to the fact that NS5A was not easily recognized by the 9E10 mAb when associated with apoptotic cell debris in the supernatants. Alternatively, it could be that a fraction of bioactive NS5A becomes liberated after interaction of the cell debris with monocytes.

### Liver NK cells paradoxically express high NKG2D levels

Given that apoptotic HCV-replicating cells seem to release NS5A, we made an effort to resolve the issue of NKG2D expression on NK cells in the infected liver. We analyzed liver-infiltrating NK cells and paired circulating NK cells in 11 additional HCV viremic patients who underwent liver biopsy for diagnostic purpose. NKG2D levels on peripheral NK cells fully matched with those in the first series of patients (MFI 59 ±7.7 and 61±15, respectively). Of note, the proportion of NK cells among liver-infiltrating mononuclear cells was very low (2.7%±0.7%), as already reported in HCV-infected livers [9], [36], [37]. To our surprise - but in line with previous observations in the rat and human ([10], [38], [39]- liver NK cells expressed higher NKG2D levels than their circulating counterpart (mean MFI 115.5 ±17.4 versus 59±7.7, P = 0.002, Wilcoxon matched-pairs test). NKG2D analysis in a representative liver sample is shown in Figure 6C (left panel) and Supplementary Figure S3. By comparison, NKG2D levels on liver NK cells from 8 control patients with non-infectious chronic inflammatory liver disease were not significantly different from those observed on circulating NK cells (mean MFI 106.2 and 87, respectively). Staining of HCV-infected liver sections showed that NKG2D+ cells were indeed very scarce even among large portal infiltrates. Only few cells were found positive in sinusoidal tracts (Figure 6C, right panel). Similar to what was observed for circulating NK cells, the proportion of liver NK cells and their NKG2D levels were not correlated with any HCV disease marker (not shown).

Whether these intrahepatic NKG2D^high^ NK cells were more functionally competent than their circulating counterpart could not be evaluated due to their too small number. In attempt to analyze NKG2D-mediated functions in a clinically relevant target cell system, we sought to use JHF1-infected Huh-7.5.1 cells. However, it turned out that these cells were not pertinent for NKG2D ligand expression studies. Not only HCV infection did not induce MIC expression on Huh7.5.1 cells, but none of the stimuli known to be potent inducers of NKG2D ligands (heat-shock, oxidative stress, γ-radiation, retinoic acid, inhibitors of histone deacetylase) was able to induce MIC surface expression on Huh7.5.1 cells (data not shown).

### IL-15 can antagonize the TGFβ-mediated modulation of NKG2D and NK cell functions

**Figure 7:**
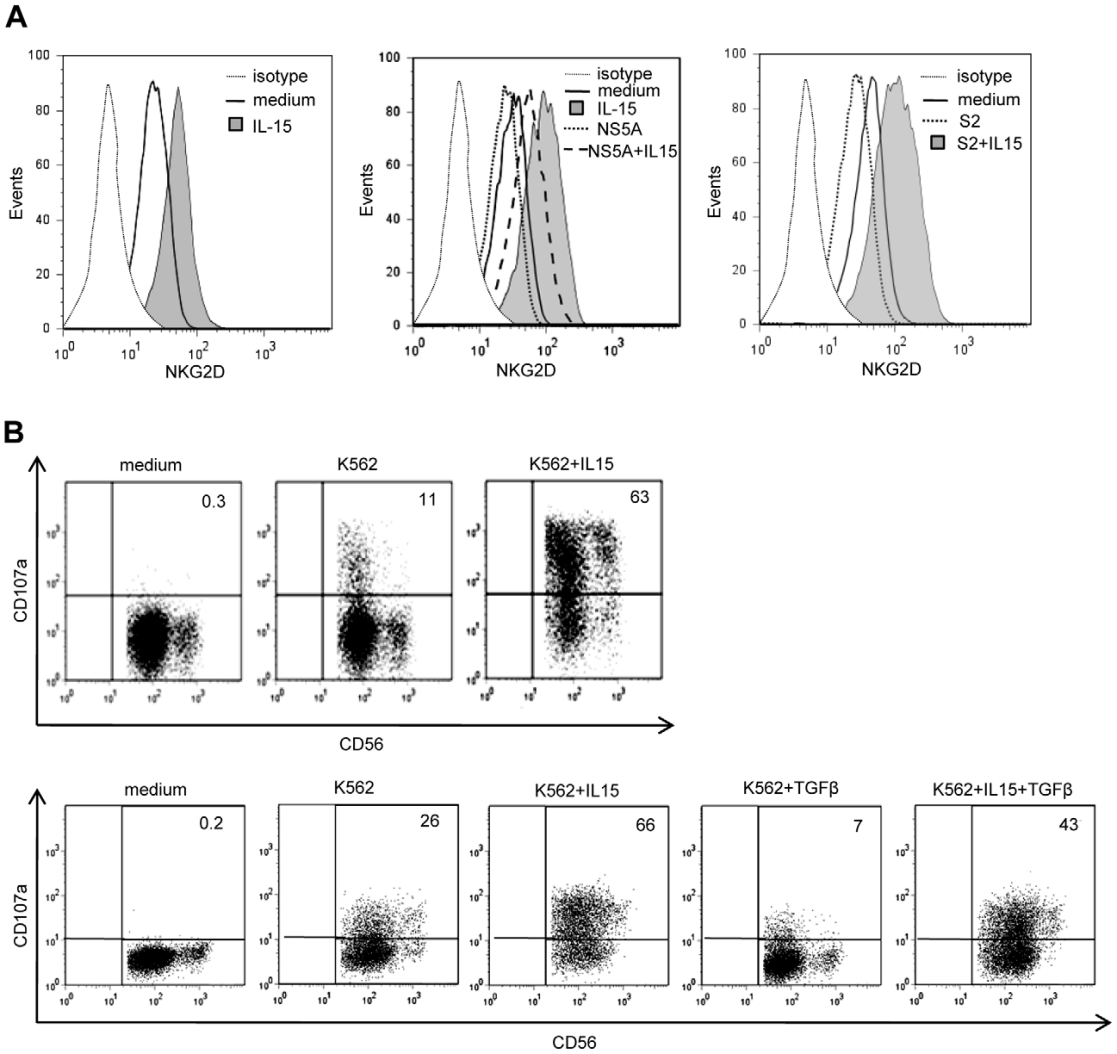
NS5A-induced alterations of NKG2D are antagonized by exogenous IL-15. A) IL-15 and TGFβ are opposite effects on NKG2D levels. *Left panel*: NKG2D staining on NK cells from one HCV patient cultured for 48 days in medium alone or in the presence of 10 ng/ml of IL-15. Results are representative of independent experiments in 3 patients. *Medium and right panels*: PBMCs from one control were cultured in the presence of 10 ng/ml of IL-15 with or without NS5A (0.5 µg/ml) or TGFβ-containing serum (S2: 32 ng/ml). Results are representative of independent experiments in 3 healthy controls. B) Purified NK cells from one HCV patient (*upper panel*) and one control (*lower panel*) were cultured for 36 hr with or without 10 ng/ml of IL-15 and/or 10 ng/ml TGFβ, after which lytic capacity to K562 target cells was measured. Numbers in the quadrant are the percentages of CD107a+ NK cells. Results are representative of 3 independent experiments.

In contrast with TGFβ, IL-15 up-regulates surface NKG2D expression [40]. Because of the reciprocal antagonism of IL-15 and TGFβ on intracellular signaling pathways [41], [42], [43], we wondered if decreased NKG2D expression on circulating NK cells from HCV patients might be amplified by a resistance of NK cells to endogenous IL-15 due to TGFβ or by a defective production of IL-15 in response to infection. PBMCs from healthy controls or HCV patients were pretreated with IL-15 for 24 hr after which NKG2D staining was performed (Figure 7A). IL-15 restored NKG2D expression on patients' NK cells at levels similar to those usually observed in controls, indicating that NK cells from HCV patients were normally responsive to IL-15. Moreover, IL-15 fully antagonized the NS5A-induced downmodulation of NKG2D on NK cells. Even in the presence of TGFβ-containing serum, IL-15 could prevent NKG2D expression. Furthermore, both NK cells from HCV patients and TGFβ-stimulated control NK cells exhibited a significant enhancement of cytotoxicity upon IL-15 stimulation (Figure 7B).

Given that pathogen components are among the stimuli that elicit production of IL-15, one could expect elevated levels of IL-15 in the serum of chronic HCV patients. IL-15 levels were not higher in patients than in controls (mean 7.61±1.93 pg/ml, and 7.98±1.40 pg/ml, respectively; ns). It must be noted, however, that IL-15 is mostly present in membrane-bound IL-15/IL-15Rα complexes [44], so that free IL-15 is unlikely to represent a reliable marker of systemic IL-15 production.

Altogether, these data suggest that overexpression of TGFβ contributes to the reduction of NKG2D and defective functions of circulating NK cells in HCV patients, a defect which can be antagonized by exogenous IL-15.

## Discussion

HCV uses a repertoire of dampening signals to subvert immune responses, a significant number of which target the innate system [45], [46]. We report here an altered expression of the NKG2D receptor as an additional HCV strategy to avoid NK-cell mediated recognition. HCV-NS5A protein, through monocyte-derived TGFβ production, downregulates expression of NKG2D on NK cells, thus reducing their cytotoxic potential and IFNγ production. Some previous studies have reported defective NK cell function in HCV infection [47], [48], [49], although others have not seen this [5], [50], [51]. Different methodologies, including the use of total PBMC or purified NK cells, fresh or cryopreserved cells, unstimulated or cytokine-stimulated cells, chromium release or flow cytometry assays, and small sample sizes, might explain why some of the findings in these studies differed from our own. We performed all experiments on freshly purified unstimulated NK cells, and measured NK cell degranulation rather than overall K562 cell lysis, because it has the advantage to shift the focus from the fate of target cells to the true response of NK cells, as previously demonstrated [52].

Different virally encoded products have been shown to impair NKG2D-mediated detection of infected cells, usually by targeting the ligands of NKG2D rather than the receptor itself [14], [16], [53], [54]. HCV only encodes a small number of structural and non-structural proteins. Consequently, each HCV gene product must have pleiotropic functions rather than highly specialized ones. Targeting NKG2D rather than its numerous ligands is at lower cost for HCV. However, it is likely that HCV must also target other receptors to escape NK cell recognition. Indeed, reduced NKp30 levels have been observed on NK cells from HCV patients [6]. Interestingly, TGFβ not only downmodulates NKG2D, but also reduces expression of NKp30 [23] and we observed a TGFβ-mediated reduction of NKp30 on control NK cells upon NS5A stimulation. This pleiotropic effect of TGFβ thus represents an economical way for HCV to shift the overall balance of NK signals towards an inhibitory phenotype. Our results are in contrast with a recent report by Oliviero et al. showing an increased proportion of NKG2D+ NK cells in HCV patients compared with healthy controls [10]. A potential explanation to this discrepancy is the surprisingly low frequency of NKG2D-positive NK cells in controls (60%) from Oliviero's study, although NKG2D is usually reported to be constitutively expressed on all NK cells [12]. In our study, the differences in patients and controls only affected NKG2D expression levels, but not the frequency of NKG2D-positive cells.

We show that TGFβ production results from NS5A interaction with the TLR4 complex on monocytes, which leads to a dysregulated equilibrium of inflammatory cytokines, i.e. increased IL-10 and defective IL-12 production. IL-10 is a potent suppressor of TLR-induced inflammatory responses, and an important target of immune subversion for some pathogens. IL-10 signaling activates STAT3, which positively regulates TGFβ promoter activity [55]. Previous studied identified that HCV core, NS3 or NS4, but not E2 protein induced monocyte-derived IL-10 production [56], [57]. In the case of core and NS3, this effect was mediated through TLR2 signaling [58]. Unfortunately, NS5A was not tested in these studies. We think that NS5A signals through TLR4 in monocytes, because preincubation with a blocking anti-TLR4 mAb inhibited NS5A-mediated IL-10 production. Also, binding experiments showed that NS5A interacted directly with TLR4 on monocytes. The likelihood of contaminating LPS contributing to the NS5A-mediated effect was ruled out by the lack of concomitant IL-12 production. The downregulation of NKG2D required direct monocyte-NK cell contacts, as it was completely lost in the Transwell system. This suggests that, in addition to produce soluble TGFβ, NS5A-stimulated monocytes might express membrane-bound TGFβ, which would further participate in NKG2D modulation through direct contact with NK cells. In support of this hypothesis, myeloid-derived suppressor cells (MDSCs), a subpopulation of immature myeloid cells with suppressor functions, were shown to downregulate NKG2D expression and inhibit liver NK cell cytotoxicity in cancer-bearing mice, through expression of membrane-bound TGFβ and direct contact with NK cells [59]. Furthermore, MDSCs were recently shown to inhibit NK cell functions through direct cell-cell contact in the context of hepatocellular carcinoma in humans [60], [61]. Whether a subpopulation of NS5A-binding monocytes characterizes MDSCs able to suppress NK cell activity through TGFβ production is under investigation in our laboratory. Further studies will be needed to determine the role of MDSCs in HCV-infected patients.

The effect of NS5A on monocytes is reminiscent of other proteins from persistent viruses. Interaction of HTLV-1 p30 protein with TLR4 signaling stimulates the release of IL-10 and hampers the release of pro-inflammatory cytokines from macrophages [25]. HIV Tat-induced IL-10 production by monocytes is regulated by p38 MAPK [62]. The LMP1 protein of EBV also induces IL-10 via p38 and PI3 kinase activation [63]. The vaccinia virus A52R protein activates p38 and JNK, and promotes TLR4-induced IL-10 production, while inhibiting NFkB-dependent genes IL-8 and RANTES [64]. Human major group rhinoviruses [26] downmodulate the accessory function of monocytes by inducing IL-10 production and inhibiting IL-12 production. Together, those reports and our findings open the idea that engagement of TLR4 may generate negative signals that are necessary for immune subversion and viral persistence.

NS5A is localized in the perinuclear regions of the infected cell, but is not present in the circulating virions. However, there is now increasing evidence that HCV mediates hepatocyte apoptosis [31], [32], [33], [65], [66], which may allow HCV proteins to be released in the extracellular milieu. We observed that supernatants of Huh7.5.1 human hepatoma cells transfected with the JFH1 infectious replicon reproduced the effect of recombinant NS5A on NKG2D downmodulation, suggesting that NS5A might be released in the culture medium from apoptotic cells. However, direct proof for the presence of NS5A protein in HCV-infected cell supernatants is still lacking, as it was not accessible by anti-NS5A mAb in Western blot or depleting experiments. The possibility that a fraction of bioactive NS5A only becomes liberated after interaction of the cell debris with monocytes is supported by our observation of a comparable NS5A binding on monocytes of recombinant protein and of supernatants from JFH1-replicating Huh7.5.1 cells.

To reconcile this idea with our finding that the rare NK cells present in HCV-infected liver expressed high NKG2D levels, one may envision a scenario in which the local cytokine microenvironment of the liver sinusoids, in particular IL-15 (which is produced by Kupffer cells) can inhibit the effect of TGFβ and enhance NKG2D expression. Indeed, IL-15 antagonizes the TGFβ immunosuppressive effects through blockade of the Smad3 signaling pathway [41], [42], [43]. Expression of IL-15 within HCV-infected livers was reported to show a sinusoidal distribution [67]. We found that the rare intrahepatic NKG2D-positive cells were located in sinusoidal tracts, but not in parenchymatous areas or necroinflammatory lesions where NS5A release by apoptotic infected cells is likely to occur. Moreover, we showed that IL-15 could fully prevent the TGFβ-mediated modulation of NKG2D and NK cell functions in vitro. A possibility is that once having migrated to areas of inflammation in the liver, most NK cells would be induced to apoptosis. This phenomenon might be favored by the abnormal expression of the Programmed-Death 1 (PD-1) molecule on NK cells, which has been observed in chronically-infected HCV patients [68]. Only NK cells expressing high levels of NKG2D would preferentially home to the liver, or could survive in the liver due to their resistance to apoptosis under inflammatory conditions. This hypothesis is the matter of current investigation in our laboratory. That NKG2D levels, either on peripheral or liver-infiltrating cells, were not correlated with virological or histological markers of the liver disease has also been observed by others [39] and might reflect such complex interactions. Unfortunately, our attempt to clarify whether increased NKG2D expression on liver NK cells is associated with enhanced cytotoxic activity was hindered by the highly restricted access to fresh liver biopsy tissue from chronically infected patients. The availability of noninvasive biomarkers for first-line assessment of liver fibrosis has led to a dramatic decrease in the use of liver biopsy for patients with chronic hepatitis C. Regrettably, functional analysis of liver-infiltrating immune cells in the few patients still undergoing liver biopsy is probably not representative of natural HCV infection.

Altogether, our observations raise the idea that reducing IL-10 and/or TGFβ bioavailability could be a suitable means to restore NK cell functions in chronic hepatitis C. However such approach could dangerously modify the overall equilibrium between effector and regulatory mechanisms. Rather, we propose the use of IL-15 - or biologically active soluble IL-15/IL-15Rα complexes [69] - as an adjuvant therapeutic agent to restore NKG2D-mediated NK cell functions. Notably, the pathways triggered by IL-15 receptor signaling are required for the NKG2D-mediated signal transduction and cytotoxicity [70]. Jinushi et al. showed that dendritic cells (DCs) from HCV-infected patients have impaired IL-15 production upon stimulation by IFNα [71]. Given the role of NK cells in promoting optimal initiation of adaptive CD8 T cell responses, and the role of IL-15 in the proliferation and survival of NK and CD8 T cells, IL-15 might help not only in establishing strong innate responses, but also in inducing more robust antiviral CTL responses.

## Materials and Methods

### Subjects

The HCV viremic patient group consisted of 34 chronically infected patients (anti-HCV antibodies and HCV RNA positive) who were naive of treatment, or who discontinued treatment at least 6 months before study. The HCV aviremic group was composed of 9 subjects with sustained viral response (SVR) following IFNα and/or ribavirin therapy, with viremia remaining undetectable for at least 6 months at the time of study. The main clinical characteristics of the patients are shown in Table 1. Patients with primary biliary cirrhosis (n = 4) or autoimmune hepatitis (n = 5) were used as non-infectious chronic inflammatory liver disease controls. An additional series of 11 HCV viremic patients was studied for paired analysis of circulating and liver-infiltrating mononuclear cells. The control group consisted of 23 age and sex matched blood donor volunteers seronegative for HCV.

**Table 1:** Main characteristics of the patients.

Age (years)*		56±2.5 (20–79)
Gender (M/F)		18/16
HCV genotype (n, %)	1	22 (64.7)
	2 and 3	2 (5.9)
	4	6 (17.6)
	5	4 (11.8)
Viral load (kUI/ml)*		1,169±342 (0.6–8,000)
ALT (xN)*		1.6±0.15 (1–5)
Liver fibrosis stage (n, %)	0–1	18 (53)
	2	5 (14.7)
	3	5 (14.7)
	4	6 (17.6)
Liver activity score (n, %)	0–1	26 (76.5)
	2	2 (5.9)
	3	6 (17.6)

*expressed as mean values ± standard error (range).

### Ethics statement

The study was performed in accordance with the Declaration of Helsinki and French legislation, and received approval of the Grenoble University Hospital ethical committee (03/APTF/1). All study participants provided written informed consent.

### Flow cytometry

Blood samples were processed within 2 h of collection and PBMCs were separated by Lymphoprep gradient centrifugation (Biowest). NK cells or monocytes were freshly purified from PBMCs by negative selection using magnetic microbead separation kits (Miltenyi Biotec) with purity higher than 90%. Liver-infiltrating mononuclear cells were isolated from fresh biopsy as reported [72] and processed immediately for staining and flow cytometry. Cells were incubated for 20 min at 4°C with combinations of the following antibodies: CD3-FITC, CD56-PE, CD8- or CD4-PerCP (BD Biosciences); NKG2D-APC or isotype-matched control antibodies of irrelevant specificity (BD PharMingen). Cells were fixed in 1% formaldehyde and analyzed on FACSCalibur (BD Biosciences), collecting a total of 100,000 events in a live gate, and data were analyzed using FlowJo software.

### NK cell degranulation assay and IFNγ production

NK cell cytototoxic potential was studied using CD107a as a marker of degranulation. Freshly isolated NK cells were incubated in the presence or absence of K562 cells, C1R cells or C1R-MICA transfectants (a generous gift from A. Toubert, Hopital St-Louis, Paris, France) as target cells. CD107a-Pe-Cy5 antibody (BD) was added directly to the tubes at 20 µg/mL. After 1 hour at 37°C in 5% CO2, brefeldin A (10 µg/ml, Sigma) and monensin (6 µg/ml, Sigma) were added for additional 5 hr, and cells were stained with CD3-FITC and CD56-PE antibodies, fixed and analyzed by flow cytometry. Where indicated, NKG2D blocking antibody (20 µg/ml, Coulter Immunotech) was added. For intracellular IFNγ analysis, NK cells were incubated for 6 hr with K562 cells, fixed following staining with anti-CD3 and anti-CD56, permeabilized with 0.2% saponin and stained with IFNγ-PE antibody (BD) for an additional 30 min.

### Cell cultures and reagents

Recombinant TGFβ and IL-15 were purchased from R&D Systems. Cytokine levels were quantified using ELISA (TGFβ, IL-10 and IL-15 quantikine kits from R&D Systems; IL-12 ELISA kit from Diaclone). Soluble MICA was measured in the sera with a sandwich ELISA as described [73]. Recombinant soluble MICA was consistently detected at concentration of 0.2 ng/ml.

The following genotype 1a-derived recombinant HCV proteins were used: E. Coli-derived full length core, NS3, NS4 and NS5 (Axxora LKT). In confirmatory experiments, we used E. Coli-derived rNS5A amino acid 2061–2392 (Axxora LKT) and yeast-derived rNS5 2054–2995 (ibtsystems). Recombinant HCV-E2 protein (Immunodiagnostics) was purified from baculovirus-infected insect cells. β2microglubulin was used as control for E. Coli-purified protein. All proteins were used at a final concentration of 0.1 to 1 µg/ml. Endotoxin levels determined by the limulus amebocyte lysate assay (BioWhittaker Cambrex) were between 0.05 and 0.2 endotoxin unit/µg protein (0.054 EU/µg for the full length NS5A protein from Axxora LKT used in most experiments). To ensure that trace amount of endotoxin did not contribute to the observed responses, rNS5A was subjected to polymyxin B (10 µg/ml) (Sigma–Aldrich, St. Louis, MO, USA) for 15 min at room temperature.

For blocking experiments, cells were incubated with 10 µg/ml of neutralizing mAb to TLR4, TLR2 or CD14 (eBioscience), soluble IL-10 receptor, anti-IL-10 neutralizing antibody (R&D Systems) before the addition of HCV protein. Isotype-matched antibodies were used as controls (Coulter Immunotech). Inhibitors of the signaling molecules JNK (SP600125), p38 (SB203580), PI3 kinase (LY294002), and MEK1 (U0126) were from Calbiochem.

### In vitro system of HCV replication

Huh-7.5.1 cells were kindly provided by Pr. Francis V. Chisari (The Scripps Research Institute, La Jolla, CA), and grown in Dulbecco's modified Eagle's medium-based medium as described [35]. Productive HCV infection was achieved as described [34], [35]. Briefly, Huh-7.5.1 cells were transfected with genomic HCV RNA transcribed in vitro from the plasmid pJFH1 [34] (a kind gift from Takaji Wakita, National Institute of Infectious Diseases, Tokyo, Japan) used as template, and cells were then passaged when necessary to maintain subconfluent cultures throughout the experiment. Cultures were probed for the frequency of HCV protein-expressing cells by in situ immunofluorescence, and infectivity titers in culture supernatant were assessed by focus-formation assay [35].

For binding experiments, monocytes were incubated for 30 min at 4°C with supernatants from non-infected or JFH1-replicating Huh-7.5.1 cells, or with 0.5 µg/ml of recombinant NS5A (positive control) or NS4 (negative control). Where indicated, culture supernatants were passed through a 0.45-µm filter following low-speed centrifugation to remove cellular debris.

After washing and blocking with human IgG, cells were incubated for 40 min at 4°C with the mouse 9E10 mAb specific for genotype 2a NS5A (a generous gift from C.M. Rice, Rockefeller University, NY, USA) [74], followed with PE-labeled goat anti-mouse Ig, and analyzed by flow cytometry.

### Immunostaining of liver tissue

Expression of MIC was evaluated on liver biopsy samples submitted to the Department of Pathology for diagnostic purpose. Paraffin-embedded liver biopsy sections (12 patients) were stained with anti-MIC (clone SR99 [75]) or anti-NKG2D (R&D Systems) mAb, followed with biotinylated goat anti-mouse Ig. For double immunofluorescence staining, cryosections (3 patients) were stained overnight at 4°C with anti-HCV-NS5A mAb (clone 7-D4, BioDesign), followed with FITC-labeled goat anti-mouse IgG, then incubated with biotinylated anti-MIC mAb, followed with streptavidin-Cy3. Slides were mounted with DAPI-containing medium (Vector Laboratories) and analyzed by immunofluorescence (Eclipse E888, Nikon) or confocal laser scanning (TCS SPS AOBS model, Leica) microscopy.

### Statistical analysis

All statistical tests were performed using Stata software (version 8.0). Qualitative values between groups were compared using the chi-square test or Fisher's exact test, and quantitative values were compared using the non-parametric Mann-Whitney U test. The Wilcoxon test was used to compare matched pairs. Correlation between two variables was determined using Spearman's coefficient (rho). Two-sided P values less than 0.05 were considered significant.

### Accession numbers

NKG2D, P26718 (NKG2D_HUMAN); Toll-like receptor 4, O00206 (TLR4_HUMAN); NS5 protein, Q81596 (Q81596_9HEPC).

## Supporting Information

Figure S1 CD107a expression on NK cells is positively correlated with NKG2D levels (Spearman rho (*r*)  = 0.62, P = 0.008).
(4.29 MB TIF)

Figure S2 HCV-NS5A protein downregulates NKp30 levels on NK cells. Control PBMCs (0.2×10^6^/ml) were cultured in medium alone (white bars) or in the presence of 0.5 µg/ml HCV-NS5A protein (black bars) for 48 h, and NKp30 expression (MFI) was analyzed on CD3-CD56+ NK cells. Mean ± SEM values in 4 healthy controls. * P<0.01.
(0.72 MB TIF)

Figure S3 NKG2D analysis in a representative liver sample. Freshly isolated liver infiltrating lymphocytes were stained with mAb to CD3, CD56 and NKG2D, or isotype control and analyzed by flow cytometry. A) Isotype control, B) CD3+ CD56- T lymphocytes, C) CD3-CD56+ NK cells.
(0.06 MB TIF)
