# Supplementary figures and images for: Hepatitis C Virus (HCV) Evades NKG2D-Dependent NK Cell Responses through NS5A-Mediated Imbalance of Inflammatory Cytokines
Source: PLoS Pathog. 2010 Nov 11;6(11):e1001184. doi: 10.1371/journal.ppat.1001184 (PMC2978723; doi:10.1371/journal.ppat.1001184)

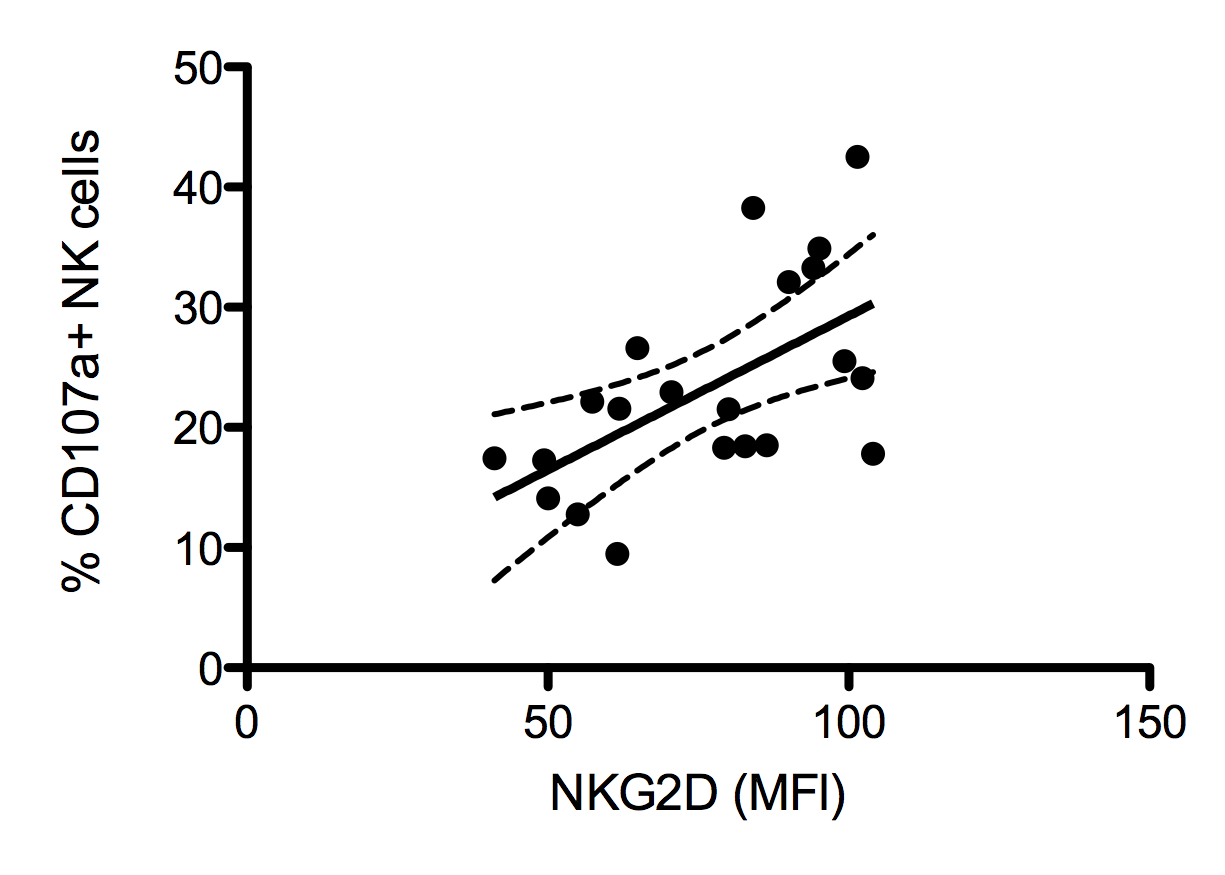

Supplement: Figure S1 — CD107a expression on NK cells is positively correlated with NKG2D levels (Spearman rho (r) = 0.62, P = 0.008). (4.29 MB TIF) [file ppat.1001184.s001.tif]

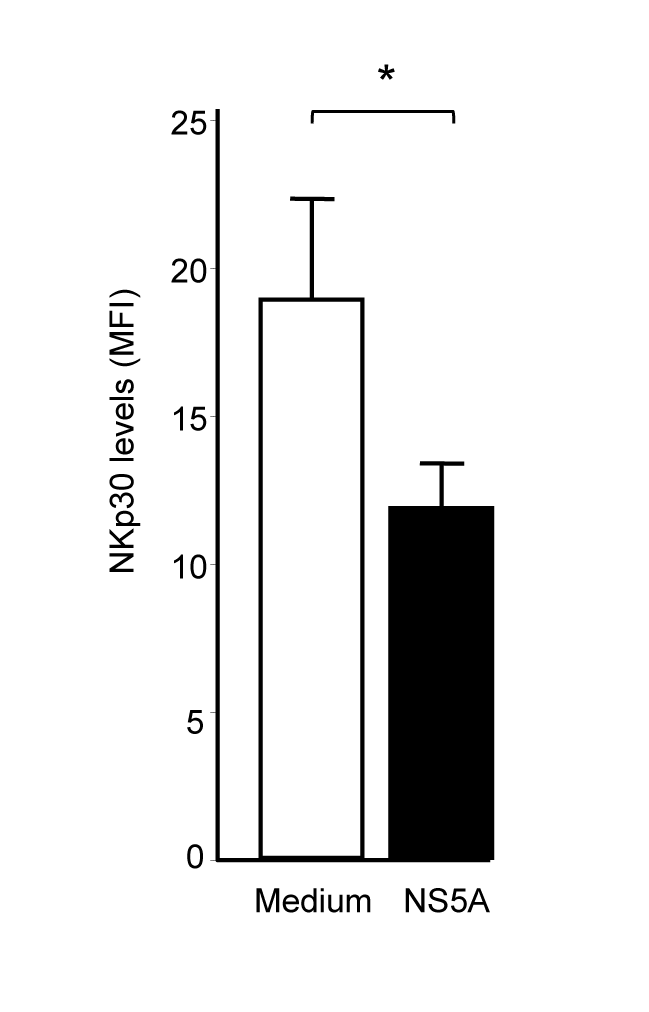

Supplement: Figure S2 — HCV-NS5A protein downregulates NKp30 levels on NK cells. Control PBMCs (0.2×10^6^/ml) were cultured in medium alone (white bars) or in the presence of 0.5 µg/ml HCV-NS5A protein (black bars) for 48 h, and NKp30 expression (MFI) was analyzed on CD3-CD56+ NK cells. Mean ± SEM values in 4 healthy controls. * P<0.01. (0.72 MB TIF) [file ppat.1001184.s002.tif]

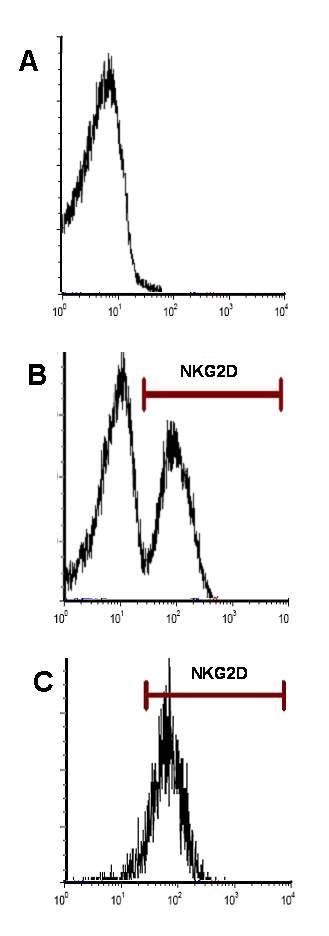

Supplement: Figure S3 — NKG2D analysis in a representative liver sample. Freshly isolated liver infiltrating lymphocytes were stained with mAb to CD3, CD56 and NKG2D, or isotype control and analyzed by flow cytometry. A) Isotype control, B) CD3+ CD56- T lymphocytes, C) CD3-CD56+ NK cells. (0.06 MB TIF) [file ppat.1001184.s003.tif]
